# A Super-Learner Model for Tumor Motion Prediction and Management in Radiation Therapy: Development and Feasibility Evaluation

**DOI:** 10.1038/s41598-019-51338-y

**Published:** 2019-10-16

**Authors:** Hui Lin, Wei Zou, Taoran Li, Steven J. Feigenberg, Boon-Keng K. Teo, Lei Dong

**Affiliations:** University of Pennsylvania, Department of Radiation Oncology, Pennsylvania, 19104 United States

**Keywords:** Lung cancer, Lung cancer, Statistics, Statistics

## Abstract

In cancer radiation therapy, large tumor motion due to respiration can lead to uncertainties in tumor target delineation and treatment delivery, thus making active motion management an essential step in thoracic and abdominal tumor treatment. In current practice, patients with tumor motion may be required to receive two sets of CT scans – the initial free-breathing 4-dimensional CT (4DCT) scan for tumor motion estimation and a second CT scan under appropriate motion management such as breath-hold or abdominal compression. The aim of this study is to assess the feasibility of a predictive model for tumor motion estimation in three-dimensional space based on machine learning algorithms. The model was developed based on sixteen imaging features extracted from non-4D diagnostic CT images and eleven clinical features extracted from the Electronic Health Record (EHR) database of 150 patients to characterize the lung tumor motion. A super-learner model was trained to combine four base machine learning models including the Random Forest, Multi-Layer Perceptron, LightGBM and XGBoost, the hyper-parameters of which were also optimized to obtain the best performance. The outputs of the super-learner model consist of tumor motion predictions in the Superior-Inferior (SI), Anterior-Posterior (AP) and Left-Right (LR) directions, and were compared against tumor motions measured in the free-breathing 4DCT scans. The accuracy of predictions was evaluated using Mean Absolute Error (MAE) and Root Mean Square Error (RMSE) through ten rounds of independent tests. The MAE and RMSE of predictions in the SI direction were 1.23 mm and 1.70 mm; the MAE and RMSE of predictions in the AP direction were 0.81 mm and 1.19 mm, and the MAE and RMSE of predictions in the LR direction were 0.70 mm and 0.95 mm. In addition, the relative feature importance analysis demonstrated that the imaging features are of great importance in the tumor motion prediction compared to the clinical features. Our findings indicate that a super-learner model can accurately predict tumor motion ranges as measured in the 4DCT, and could provide a machine learning framework to assist radiation oncologists in determining the active motion management strategy for patients with large tumor motion.

## Introduction

Respiratory motion poses a great challenge in the treatment of lung cancer with radiation therapy^1–3^. Target and normal tissue motion can be quite complex and patient-dependent^4^. To address this issue in modern radiation therapy treatment planning, an internal margin is assigned based on the patient’s 4-dimensional Computed Tomography (4DCT) to form an Internal Target Volume (ITV)^5,6^, where the extent of the tumor motion is included. However, when the tumor motion extent is large, the ITV may possess prohibitively large volume that could lead to increased treatment toxicity. Therefore, patients with a large tumor motion are more suitable for using an active motion management strategy such as a breath-hold technique^7^ or the use of compression belt^8^ to reduce the magnitude of tumor motions due to diaphragmatic breathing^9^. These active motion management procedures, however, require extra steps in the simulation workflow since the need for an additional simulation CT scan with motion management is known only after the motion was assessed from an initial free-breathing 4DCT scan. The motivation of this work is to test the feasibility of predicting tumor motion without an initial free-breathing 4DCT scan such that these patients can be directly identified and undergo an active motion management strategy for simulation. If successful, the motion prediction can avoid extra radiation dose to the patient due to additional CT imaging. In addition, streamlined active motion management without the initial 4DCT motion assessment can be tolerated by most patients who might be too tired or too sick to perform two 4DCT procedures in the same day and increase the quality of the scan. This also potentially reduces staff and equipment resource needed for the simulation.

Previously, there were extensive studies for the characterization and prediction of lung tumor motion, such as the prediction of real-time tumor motion tracking during treatment^10–13^, the characterization of tumor motions to estimate severity of 4DCT motion artifact^14,15^, or the detection of inter-fraction motion pattern change^16^. The input of these studies was 4DCT images of patients or motion trace measured by fiducial markers. In comparison, the aim of this study is to develop a motion prediction system prior to any patient motion measurement. To achieve that, non-motion specific features such as those presented in the diagnostic CT and EHR data are used as the input. Due to potential complex relationship of these input parameters, a machine learning approach is proposed in this study to improve prediction accuracy. There were previous attempts trying to predict tumor motions based on clinical features. For example, Liu *et al*.^4^ have shown the magnitude of tumor motion in the SI direction is correlated with the lobe location where the tumor resides; however, they failed to provide accurate predictions using this single-factor correlation approach. Considering the complexity of lung motion behavior and patient-specific factors, most of the previous studies only explored limited features. This work investigates the comprehensive clinical and imaging features to build a predictive model with a goal to identify those patients who may present a large range of tumor motion and require an active motion management strategy for their subsequent radiation therapy.

In this study, we first propose a machine learning approach to investigate the inherent correlations between input features (imaging and clinical features) and tumor motion ranges. In the second step, we developed a super-learner model that employed the input features to predict the three-dimensional lung tumor motion ranges. The development and validation of the proposed model were demonstrated in an extensive patient database of 150 lung patients.

## Results

### Feature selection

**Figure 1:**
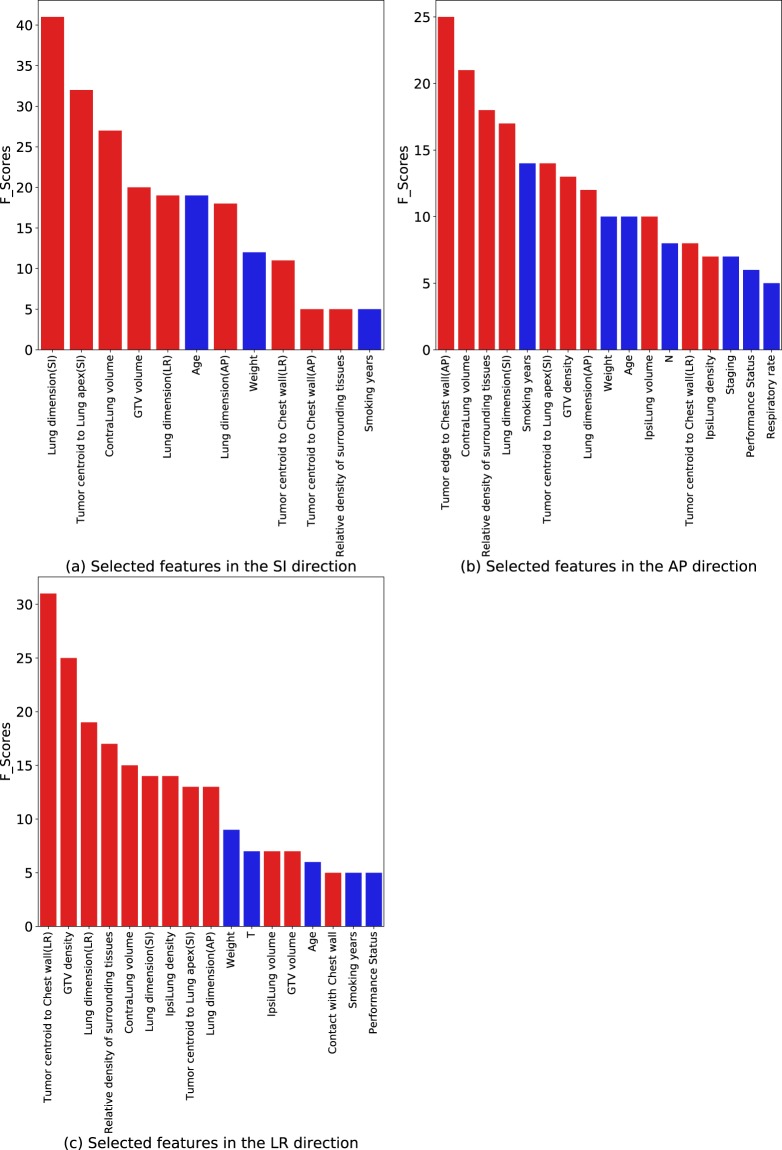
Feature importance ranks in the SI, AP and LR directions obtained by XGBoost RFE and col-linearity removal are shown in (**a**–**c**). Imaging features are plotted in red and clinical features are plotted in blue. The F-scores were averaged over ten rounds of independent tests.

The selected features with top averaged F-scores after Recursive Feature Elimination (RFE) and collinearity removal in each direction are shown in Fig. 1. For the SI-direction, 12 features out of 27 features were selected; in the AP direction, 17 features out of 27 features were selected; and for the LR-direction, 17 features out of 27 features were selected. Overall, the imaging features demonstrated a higher degree of importance in motion prediction than the clinical features. Among imaging features, the tumor centroid or edge location relative to the boundaries of lungs (chest wall or the apex of the lung), lung dimensions, and the lung volume were the top selected features. Among clinical features, the patient’s weight, age, and pack years of smoking were the most frequently selected features. During the ten rounds of independent tests, certain features consistently showed greater correlations with the tumor motion in each direction, and they are: Lung dimension (SI) and Tumor centroid distance to the lung apex (SI) for the SI direction; Distance of tumor edge to the chest wall (AP) and Contralateral lung volume for the AP direction; Distance of tumor centroid to the chest wall (LR) and GTV density for the LR direction.

### Hyper-parameter tuning

**Figure 2:**
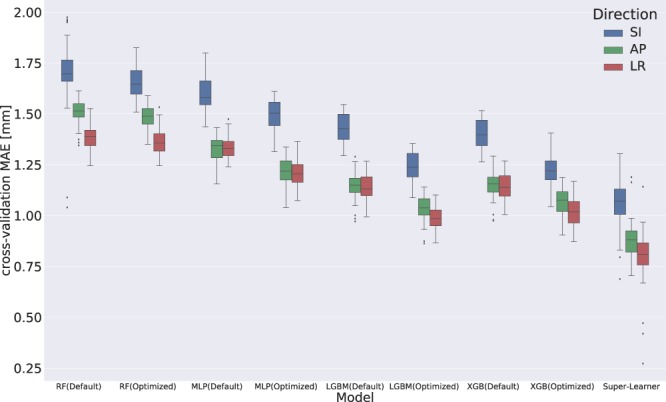
The five-fold cross-validated Mean Absolute Error (MAE) of each machine learning base model with default hyper-parameter settings, with optimized hyper-parameters and the MAE of the Super-Learner model. The benefit of hyper-parameter tuning can be demonstrated by comparing the MAE of four base models with default hyper-parameters and with optimized hyper-parameters. The base models include Random Forest (RF), Multi-Layer Perceptron (MLP) Networks, LightGBM (LGBM) and XGBoost (XGB). The power of building up super-learner models can be demonstrated by the MAE improvements between the super-learner model and each optimized base model.

All the machine learning base models utilized in this study contain several hyper-parameters that can affect performance significantly. Our experimental results allowed us to measure the extent to which hyper-parameter tuning via Bayesian optimization improved each machine learning base model’s performance compared to its baseline settings. Figure 2 compared the MAE improvements of the tuned parameters to its default settings for each base model across the dataset in all three directions. The results demonstrated why it is unwise to use default ML algorithm hyper-parameters: hyper-parameter tuning improves the model’s predictive MAE by ~2–11%.

### Super-learner model

The MAE improvements of the super-learner model in five-fold cross-validation are illustrated in Fig. 2 by comparing to the MAE of each base model with optimized hyper-parameters. As expected, the super-learner model outperformed each individual base model. The results demonstrated that the use of the super-learner model leads to approximately 4–40% decrease in MAE.

### Prediction performance of the super-learner model

**Figure 3:**
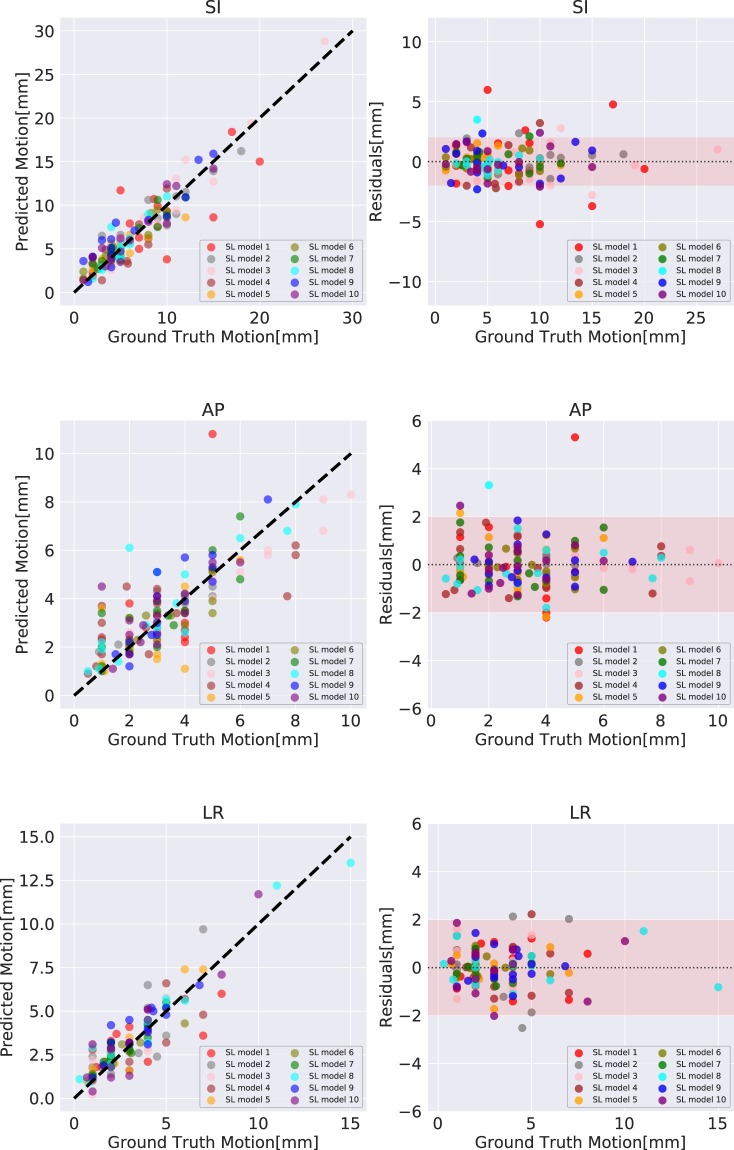
Predicted values of the super-learner models versus ground truth values in the SI, AP and LR directions and the corresponding residual plots. A 2 mm error region is highlighted in each residual plot. The independent ten test set results of each super-learner model are plotted in different colors.

The predicted tumor motion values in the SI, AP and LR direction were compared with the ground truth tumor motion ranges and illustrated by Fig. 3(a,c,e). The corresponding residual plots were shown in Fig. 3(b,d,f). A 2-mm margin, which is a typical CT slice resolution used in the patient simulation^17^, was applied to evaluate the residual errors. The MAE and RMSE of predictions in the SI direction are 1.23 mm and 1.70 mm respectively; the MAE and RMSE of predictions in the AP direction are 0.81 mm and 1.19 mm respectively; the MAE and RMSE of predictions in the LR direction are 0.70 mm and 0.95 mm respectively. To quantify the outliers, 95th and 99th percentile errors of the predictions were also calculated: in the SI direction, the 95th percentile and 99th percentile error are 2.51 mm and 3.50 mm; in the AP direction, the 95th percentile and 99th percentile error are 2.10 mm and 3.81 mm; in the LR direction, the 95th percentile and 99th percentile error are 1.65 mm and 2.35 mm.

## Discussions and Conclusion

In this work we proposed and implemented a machine learning pipeline to investigate the relationship of extensive input features and lung tumor motion ranges, and developed a super-learner model to predict the tumor motion ranges in three dimensions based on the diagnostic CT images and EHR data of the patient. To the best of our knowledge, this is the first study that introduced super-learner models to the tumor motion estimation in radiotherapy, and the built model was validated and tested on one hundred and fifty clinical cases, which is the most extensive study to date.

The study findings indicated that imaging features were more discriminative than the clinical features for the task of predicting tumor motion ranges in the SI, AP and LR direction. Specifically, the tumor location relative to the boundaries of lungs (the chest wall or the apex of the lung) and the lung dimension in each direction are recognized to have great impacts on the tumor motions, which are new features not identified by previous studies. On the aspect of clinical features, the top selected features include the patient’s weight, age and the pack years of smoking. This is consistent with clinical observations, as smoking can decrease the lung expansion and capacity^18^, the ventilation of lungs can be altered by the patient weight^19^, and the respiratory muscle strength may change along with age^20^, subsequently affecting the respiratory pattern of a patient. The features selected by this study can potentially help clinicians to decide which features can be used to estimate the lung tumor motion. The predictive accuracy of the super-learner model suggested the feasibility of utilizing a super-learner model to estimate the tumor motion ranges according to the patient CT images and EHR data, which were available prior to the 4DCT simulation. If the findings in this study can be further extended on a larger scale and reproduced in prospective studies, the super-learner model described here can optimize the current radiotherapy simulation workflow for the lung cancer patients by providing individualized motion management strategy to each patient without the assistance of an initial free-breathing 4DCT scan.

In this study, we found that XGBoost-based Recursive Feature Selection helped reduce the redundancy of inputs by lowering the number of features, thus increased the ratio between the number of input data and the number of features. However, it is necessary to assess this supervised feature selection method with external validation data to avoid over-optimistic predictive performance due to the bias of feature selection. To alleviate the overfitting problem raised by feature selection bias, the XGBoost model used for feature selection used fewer feature sampling and a shallow depth of the tree than the XGBoost model used for prediction. More importantly, the testing data was kept independent of the feature selection process. A common mistake is to involve not only the training data but also the testing data for feature selection, which causes overfitting problem due to the information leakage from the testing data during the feature selection phase. The correct procedures are deriving a subset of features only within the training data, and then inferring the predictive model based upon the selected features. During our testing phase, the pre-trained model was evaluated using independent testing data, and therefore minimized the risk of bias that might be introduced using XGBoost for feature selection.

We note some limitations of the current study that highlight opportunities for future enhancement. The first limitation is the extraction of imaging features. The current approach for extraction of the imaging features was performed manually and is very time-consuming. As these imaging features were demonstrated to be critical to the model buildup and performance, such a manual approach can benefit from the development of an automatic imaging feature extraction tool. In addition, our selection of the imaging features may be subjective and miss some important information. Some studies have indicated the solution to this problem is to introduce deep learning-based automatic feature extractor^21–24^. In the future, we plan to explore the incorporation of a Convolutional Neural Network feature extractor. The addition of automatically extracted imaging features can lead to a better representation of the patient features to obtain a more reliable prediction model.

Another limitation is the handling of tumor motion close to the motion management threshold. Tumor motion predicted around the motion management threshold needs to be taken special care, as any small error in this region can deviate the motion management decision. For example, a tumor motion of 8 mm in the SI direction was predicted to be 7.5 mm. If the motion threshold of using active motion management is also 8 mm, although the absolute predictive error is only 0.5 mm, the prediction will lead to a decision on no motion management as opposed to an active motion management strategy is needed. In future work, we plan to investigate the effects of adding adaptive margins at the threshold. We also plan to quantify and associate an uncertainty level with each tumor motion prediction to further assist the clinical motion management decisions.

## Methods and Materials

This section presents the machine learning approach and describes each step of the pipeline implemented to build and evaluate a super-learner model for tumor motion range prediction.

### Ethics, consent and permissions

The Institutional Review Board (IRB) of the Hospital of the University of Pennsylvania approved this retrospective patient study (IRB# 831407). All methods used in this study were conducted in accordance with the relevant guidelines and regulations. Considering that this is a retrospective study involving minimal risk to the privacy of the study subjects, our IRB waived the need for obtaining written informed consent from the participants.

### Proposed workflow

**Figure 4:**
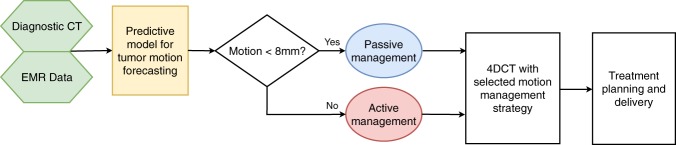
Proposed workflow of performing the automatic selection of motion management strategy prior to the patient simulation.

Figure 4 shows the high-level conceptual framework for the deployment of a super-learner predictive model in the patient simulation workflow. Compared to the current clinical practice, which requires an initial free-breathing 4DCT for tumor motion evaluation, the super-learner model can forecast tumor motion extent by utilizing imaging features extracted from the patient’s prior diagnostic CT images and the clinical features extracted from the Electronic Health Record (EHR) data. Based upon the estimated tumor motion extent, the appropriate motion management strategy can be pre-assigned to the patient for the 4DCT simulation to avoid the need for an extra 4DCT scan.

### Patient dataset collection and processing

A cohort of one hundred and fifty consecutive lung cancer patients who received proton therapy from 2014 to 2018 was retrospectively identified from the Hospital of the University of Pennsylvania. Two main categories of input features were collected: imaging features extracted from the pre-simulation diagnostic CT images (free-breathing acquisition with 3 mm slice thickness) and clinical features extracted from the EHR data. There are in total twenty-seven input features extracted from the patient data based on clinical observations and literatures^4,25^, which are summarized in Table 1. The collected output data consist of tumor motion ranges in the superior/inferior (SI), anterior/posterior (AP) and left/right (LR) directions. The tumor motion ranges were extracted from the in-house 4DCT motion evaluation records and were crosschecked by two medical physicists.

**Table 1:** Characteristics of input features.

Imaging features	Clinical features
Tumor centroid and edge locations to the apex of the lung (SI)	Age [yrs]
Tumor centroid location relative to the chest wall (AP, LR)	Weight [Lbs]
Tumor edge location relative to the chest wall (AP, LR)	Respiratory rate
Lung dimension in SI, AP and LR directions [cm]	Smoking history [pack yrs]
Tumor contact with the chest wall (2-classes)	Staging
Target lung volume [*cm*^3^]	Primary tumor (T)
Contralateral lung volume [*cm*^3^]	Regional lymph nodes (N)
Volume of Gross Tumor Volume (GTV) [*cm*^3^]	Distant metastasis (M)
GTV density [HU]	Tumor location: lung-wise (Left/Right)
Density of surrounding tissues around GTV relative to the lung	Tumor location: lobe-wise (Upper/Middle/Lower)
Target lung density [HU]	Performance status

The number of features is determined before data pre-processing.

### Machine learning pipeline

**Figure 5:**
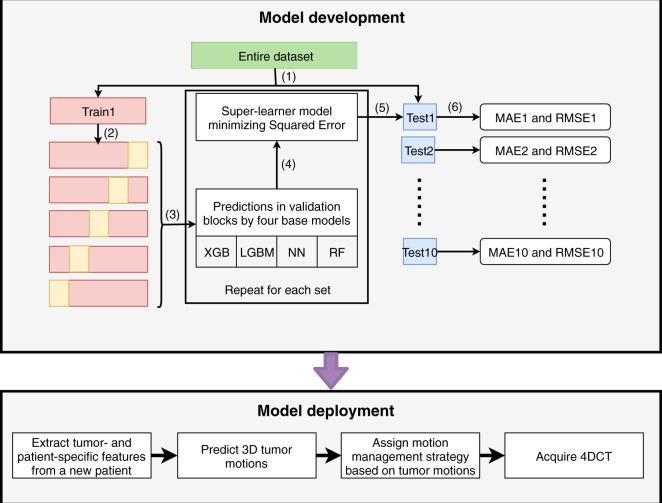
Experimental design of the Super-Learner model. The entire dataset is first divided into the training and independent test groups, in which the training group is further divided using five-fold cross-validation method (The validation set is shown in yellow).

The aim of developing a super-learner model was to learn the potential correlation of clinical and imaging features with tumor motion extent. With such a model, the tumor motion ranges in three dimensions can be predicted for a new patient and a proper motion management technique can be chosen for the patient. The study design is depicted in Fig. 5. The upper block demonstrates the model development process. The entire dataset was first divided into the training and test sets, where the test set was 10% of the dataset size and was kept independent of the entire training and validation process (step 1 in Fig. 5). The training and test sets were pre-processed independently as described in the previous section, and a subset of input features was selected from the training set (step 2). The training set was further partitioned in a five-fold cross-validation fashion, in which the entire training set was split into five sub-samples (step 3), each of which acts once as a validation set and four times as a part of a training set. Four machine learning models were selected as the base models and the optimal hyper-parameters of each model were tuned using Bayesian optimization on the training set (step 4). Once the base models were trained, a new dataset consisting of the predicted outputs of each base model on the five-fold cross-validation data and the ground-truth outputs of the cross-validation data was formed. This newly formed dataset is the basis to train the super-learner model, in which all convex combinations of base models will be evaluated and the optimal coefficients of the super-learner model will be determined by minimizing the cross-validation errors (step 5). More mathematical details of the buildup process of the super-learner model were covered in the Super-Learner Model section. The trained super-learner model was applied to the independent test set (step 6) and the Mean Absolute Errors (MAE) and Root Mean Square Errors (RMSE) of predicted values in three directions were computed. The aforementioned model building processes (step 2 to step 6) were repeated ten times to obtain MAE and RMSE using the training and test pairs produced in step 1. The model performance (MAE and RMSE) of each test set was recorded to demonstrate the true generalizability of the super-learner model. Such a super learner model can then be used to predict a new patient’s motion range in a clinical setting, which is shown in the lower block: when a new patient comes in, his/her imaging and clinical features would be extracted from the patient’s diagnostic CT images and EHR data, and were used as the inputs of the pre-trained super-learner model to predict the tumor motion extents.

### Feature selection

Feature selection methods involved in this study include Model-based Recursive Feature Elimination (RFE) and Feature Collinearity Removal.

Model-based RFE is a greedy algorithm based on the feature ranking technique. According to a specific feature ranking standard, RFE starts from a complete set and then eliminates the least relevant feature one by one to select the most important features. In this study, XGBoost was employed as the feature selection model, and the processes of XGBoost–RFE, a feature selection method that combines XGBoost and RFE, were shown as follow.

1. Input: The training set was denoted as ${{\boldsymbol{X}}}_{{\bf{0}}}={[{{\boldsymbol{x}}}_{{\bf{1}}},{{\boldsymbol{x}}}_{{\bf{2}}},\ldots ,{{\boldsymbol{x}}}_{{\boldsymbol{K}}}]}^{T}$, in which each ${{\boldsymbol{x}}}_{{\boldsymbol{i}}},i=1,2,\ldots ,K$ covered a set of m features ${\boldsymbol{P}}=[{p}_{1},{p}_{2},\ldots ,{p}_{m}]$. The output values of tumor motions were denoted as ${\boldsymbol{y}}={[{{\boldsymbol{y}}}_{{\bf{1}}},{{\boldsymbol{y}}}_{{\bf{2}}},\ldots ,{{\boldsymbol{y}}}_{{\boldsymbol{K}}}]}^{T}$, where K is the number of training patients.
2. Output:The feature rank ***R***, which was initialized as null at the initial point.
3. Repeat the following steps m times:

Train the XGBoost model and rank the features in the input feature set ***P*** by minimizing the mean absolute error;

Find out the least important features *f*;

Update the list of feature rank ***R***;

Exclude the feature with minimum criterion: ***P*** = ***P*** − ***P***(*f*).

The RFE selection is basically a recursive process that ranks features according to the measure of importance. The recursion is needed because for certain measures the relative importance of each feature can change substantially when evaluated over a different subset of features during the step-wise elimination process (in particular for highly correlated features). After the optimal subset of features was selected, the Pearson correlation heatmap was employed to discover the col-linearity among the features, in which features with high col-linearities were identified by the eigenvalues of the heatmap and were removed, as the existence of highly correlated features may reduce the predictive power of machine learning models. The feature importance was evaluated by the F-score^26,27^, which is based on the frequency of a feature being selected for the tree splitting, scaled by the squared improvement to the model and averaged over all trees. The larger the F-score, the more discriminative the feature is. The RFE and collinearity removal process was conducted in each round of independent tests, and the relative importance of each feature was measured by the F-score.

### Super-learner model

Super-learner model, also known as the model ensemble, refers to a loss-based learning method that has been proposed and analyzed by van der Laan *et al*.^28^. It addressed a common task in data mining, which is the estimator selection for prediction. In the tumor motion prediction task, it is feasible to create a set of machine learning models estimating the tumor motions in the SI, AP and LR directions but with function varieties. Specifically, each machine learning model is an estimator that mapped the input feature dataset of K patients $({{\boldsymbol{x}}}_{{\boldsymbol{k}}},{{\boldsymbol{y}}}_{{\boldsymbol{k}}})$, $k=1,2,\ldots ,K$ into a prediction function $F({\boldsymbol{y}}|{\boldsymbol{x}})$ that can be used to map an input ***x*** into a predictive value ***y***. The model selection was not limited to select only a single model. Recent studies have demonstrated that the predictive accuracy of an ensemble of multiple models can outperform a single model. One of the ensemble techniques is the super-learner modeling, which was related to the stacking algorithm introduced in neural networks by Wolpert^29^ and adapted to the regression model by Breiman^30^. The stacking algorithm was evaluated in LeBlanc and Tibshirani^31^ and the relationship to the mixed model method of Stone^32^ was discussed.

Our development of a super-learner model involved two major processes. The first step was to select and train a collection of base machine learning models. The base models may range from a simple regression model to a multi-step model involving feature screening and hyper-parameters optimization. The second step was to build up the super-learner model upon the trained base models and minimize the cross-validation risks. Specifications of each step were illustrated in the following sub-sections.

### Base machine learning models

Four classical machine learning models were selected to work as the base models in this study. Table 2 summarized each model’s characteristics. All of the base models were implemented using Python 3.6 with the scikit-learn package^33^ and Keras API^34^.

**Table 2:** Summary of the base machine learning models used in this study.

Model	Characteristics	Parameters
Random Forest^39^	A large number of decision trees based on random subsampling	n_estimators, max_depth, max_features, min_samples_split, min_samples_leaf
Multi-Layer Perceptron (MLP) Networks^40^	Auxiliary features are generated by each layer; a high number of tunable weights	layer compositions, number of hidden units, dropouts, learning rate, number of epochs
XGBoost^41^	A variation of boosting; generalizes weak learners by allowing optimization of the differentiable loss function	max_depth, min_child_weight, subsample, colsample_bytree, learning rate, num_boost_round
LightGBM^42^	Gradient boost based on the decision tree algorithm	num_leaves, feature_fraction, lambdas, max_depth, min_child_samples

### Super-learner model

The procedure of building up and training the super-learner model can be illustrated as follows. The library of base models was consisted of aforementioned four base models. Each base model is trained in a five-fold cross-validation fashion, where the validation samples of each fold are denoted as ***V***(***v***) and the training samples of each fold are denoted as ***L***(***v***) (*v* = 1, 2, …, 5). For the *v*–*th* fold, each base model was fit on ***L***(***v***) and the predictions on the corresponding validation data are denoted as ${\hat{y}}_{n,{\boldsymbol{L}}({\boldsymbol{v}})}({{\boldsymbol{X}}}_{{\boldsymbol{i}}})$, $n=1,2,\mathrm{..},4$, ${{\boldsymbol{X}}}_{{\boldsymbol{i}}}\in {\boldsymbol{V}}({\boldsymbol{v}})$.

The second step is to stack the predictions from each model to create a prediction matrix $$z=\{{y}_{n,{\boldsymbol{L}}({\boldsymbol{v}})}({{\boldsymbol{X}}}_{V(v)}),$$$$n=1,2,\ldots ,4,v=1,2,\ldots ,5\}$$, where we used the notation ***X***_*V*(*v*)_ for the input feature vectors of the validation sample. A cohort of weighted combinations ***m***(***z***|***β***) of all candidate models are proposed and indexed by a weight vector ***β***, where

1 $${\boldsymbol{m}}({\boldsymbol{z}}|{\boldsymbol{\beta }})=\mathop{\sum }\limits_{n=1}^{4}\,{{\boldsymbol{\beta }}}_{n}{{\boldsymbol{y}}}_{n,{\boldsymbol{L}}({\boldsymbol{v}})}({{\boldsymbol{X}}}_{V(v)}),\mathop{\sum }\limits_{n=1}^{4}\,{{\boldsymbol{\beta }}}_{n}=1$$

The third step is to determine ***β*** that minimizes the cross-validated errors by calculating the squared difference between the weighted vector combinations and the ground truth output ***Y***_***k***_ through

2 $$\hat{{\boldsymbol{\beta }}}=\mathop{{\rm{argmin}}}\limits_{{\boldsymbol{\beta }}}\,\mathop{\sum }\limits_{k=1}^{K}\,{({{\boldsymbol{Y}}}_{{\boldsymbol{k}}}-{\boldsymbol{m}}({{\boldsymbol{z}}}_{{\boldsymbol{k}}}|{\boldsymbol{\beta }}))}^{2}$$

Finally, the optimal weight vector $\hat{{\boldsymbol{\beta }}}$ was combined with ***y***_***n***_(***X***) based on the weights ***m***(***z***_***k***_|***β***) to create the predictions obtained by the final super-learner ***y***_*SL*_(***X***), where

3 $${{\boldsymbol{y}}}_{SL}({\boldsymbol{X}})=\mathop{\sum }\limits_{n=1}^{4}{\hat{{\boldsymbol{\beta }}}}_{n}{{\boldsymbol{y}}}_{{\boldsymbol{n}}}({\boldsymbol{X}})$$

### Bayesian optimization for hyper-parameter tuning

The performance of machine learning methods depends crucially on hyper-parameter settings and thus on the method used to select hyper-parameters. Recently, Bayesian optimization methods^35^ have been shown to outperform established methods for this problem^36^. In this study, we utilized Bayesian optimization to construct a probabilistic model to select subsequent hyper-parameter configurations. In order to select its next hyper-parameter configuration using the probability model, Bayesian optimization used an acquisition function that relied on the predictive distribution of the probability model at arbitrary hyper-parameter configurations to quantify how useful knowledge about the hyper-parameter configuration would be. The acquisition function used in this study is the expected improvement^37^ over the best previously observed function value attainable at a hyper-parameter configuration^38^. Among existing Bayesian optimization algorithms, the major difference is the model classes being used. In this paper, we empirically chose Tree Parzen Estimator (TPE)^36^.

### Metrics of predictive performance

Mean Absolute Error (MAE) and Root Mean Square Error (RMSE) were used to evaluate the predictive performance of the super-learner model. The evaluation criteria indicated the model’s prediction ability by comparing the tumor motion ranges in the testing set with their corresponding predicted values generated by the super-learner model.

MAE is a measure of the magnitude of errors, and can be calculated by

4 $$MAE=\frac{1}{K}\,\mathop{\sum }\limits_{k=1}^{K}\,|{{\boldsymbol{y}}}_{{\boldsymbol{k}}}-{\hat{{\boldsymbol{y}}}}_{{\boldsymbol{k}}}|$$

where ***y***_***k***_ is the ground truth tumor motion extents of *k*-th patient, and ${\hat{{\boldsymbol{y}}}}_{{\boldsymbol{k}}}$ is the predicted tumor motion extents, and *K* is the number of investigated patients.

RMSE is calculated by taking the square root of the mean of the square of all the errors. RMSE represents the standard deviation of the differences between predicted values and ground truth values. The effect of each error on RMSE is proportional to the size of the squared error. Consequently, RMSE is more sensitive to outliers. RMSE can be expressed by

5 $$RMSE=\sqrt{\frac{1}{K}\,\mathop{\sum }\limits_{k=1}^{K}\,{({{\boldsymbol{y}}}_{{\boldsymbol{k}}}-{\hat{{\boldsymbol{y}}}}_{{\boldsymbol{k}}})}^{2}}$$

## Data Availability

The patient datasets used for the model building and evaluation in the current study are available from the corresponding author on reasonable request and subject to Institutional Review Board (IRB) approval.
